# Reln Haploinsufficiency Enhances Fentanyl‐Induced Locomotion and Dorsal Striatal Activity Without Affecting Opioid Reinforcement and Relapse‐Like Behavior

**DOI:** 10.1111/gbb.70072

**Published:** 2026-10-06

**Authors:** Carl Litif, Avraham M. Libster, Shane Desfor, Tanieng Huang, Leanne Liaw, Annika Cheng, Francesca Telese

**Affiliations:** ^1^ Department of Psychiatry, School of Medicine University of California San Diego California USA

## Abstract

The *Reln* gene encodes the extracellular glycoprotein Reelin that regulates synaptic plasticity and activity‐dependent gene expression with implication in several neuropsychiatric disorders, including substance use disorder. Although reduction of *Reln* expression has been associated with altered responses to psychostimulants and cannabinoids, its role in opioid‐evoked behaviors has not been investigated. Here, we tested whether *Reln* haploinsufficiency alters behavioral and molecular responses to the synthetic opioid fentanyl. Heterozygous Reeler (*Reln*+/−) mice and wild‐type littermates were assessed using complementary contingent and non‐contingent models of fentanyl exposure, including multi‐phase fentanyl intravenous self‐administration paradigm, conditioned place preference paradigm, locomotor assays, and dorsal striatal immediate early gene expression. *Reln* haploinsufficiency did not alter acquisition, extinction, or cue‐induced fentanyl seeking during self‐administration, indicating stable opioid reinforcement and relapse‐like behavior. Following acute, passive fentanyl exposure, *Reln*+/− mice exhibited enhanced fentanyl‐induced locomotion and Fos immunoreactivity in the dorsal striatum, while fentanyl conditioned place preference remained unchanged. Together, these findings demonstrate that *Reln* haploinsufficiency does not modify opioid reinforcement or cue‐driven drug seeking but enhances acute drug‐evoked neuronal activation in the dorsal striatum. These results identify *Reln* as a modulatory factor in opioid‐responsive neural circuits rather than in the associative learning processes underlying opioid reinforcement.

## Graphical abstract

*Reln* haploinsufficiency does not alter fentanyl self‐administration, relapse‐like seeking, or conditioned place preference in mice. Instead, reduced *Reln* expression selectively enhances acute fentanyl‐induced locomotion and dorsal striatal Fos activation, positioning Reelin as a modulator of opioid‐evoked neuronal activity rather than opioid reinforcement.

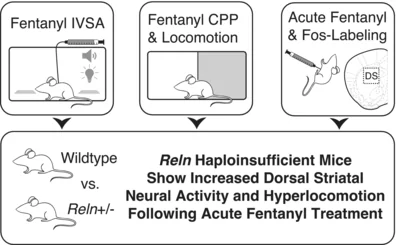

## 1 Introduction

Opioid use disorder (OUD) affects millions globally [1, 2] and is characterized by chronic drug intake, relapse vulnerability, and maladaptive learning processes involving the encoding of drug‐associated cues and contexts [3]. These behaviors reflect molecular and cellular perturbations within corticostriatal circuits, particularly the dorsal striatum, which supports action‐selection, habit‐formation, and cost–benefit valuation of reward‐seeking behaviors [4]. Opioids, including the highly potent synthetic agonist fentanyl, robustly engage striatal signaling pathways that regulate synaptic plasticity, neuronal excitability, and immediate early gene (IEG) expression [5]. Although substantial progress has been made in defining opioid‐induced behavioral adaptations, the molecular regulators that drive these plasticity processes are not clearly understood.

Reelin (*Reln*) is a large extracellular glycoprotein [6] expressed mainly in the striatum, cortex, and cerebellum [7]. While best known for its developmental role in neuronal migration, Reelin is increasingly recognized for its functions in adult neuronal plasticity through modulation of IEG expression [8, 9], dendritic spines regulation [10, 11, 12, 13], and long‐term potentiation [14, 15] in the adult brain. In humans, altered RELN expression has been implicated in several neuropsychiatric disorders characterized by disrupted synaptic plasticity [16], including autism [17, 18], schizophrenia [19, 20], Alzheimer's disease [21, 22], and attention‐deficit/hyperactivity disorder [23].

Emerging preclinical evidence also links Reelin signaling to phenotypes associated with substances of abuse. Experimental reduction of *Reln* expression in mice modifies behavioral and cellular responses to cannabinoids [24, 25, 26] and psychostimulants [27, 28, 29], including drug‐induced locomotion, reward‐related learning, and striatal activation patterns. In parallel, clinical studies report associations between altered RELN expression and phenotypes associated with alcohol use disorder [30, 31]. Together, these findings suggest that Reelin signaling may influence how drug experiences are encoded within reward‐related neural circuits.

Despite these advances, *Reln* expression in the context of opioids, much less opioid‐related behaviors, has not been investigated. Opioids differ from psychostimulants and cannabinoids in both receptor mechanisms, yet share aspects of circuit recruitment for striatal neuronal pathways involved in reinforcement learning and adaptive responding to changing reward contingencies [32]. Given the established role of Reelin signaling in regulating activity‐dependent transcription and synaptic plasticity, alterations in this signaling are well positioned to modulate opioid‐evoked neuronal activation and behavioral adaptation. To address this gap, we evaluated heterozygous Reeler (*Reln*+/−) mice and wild‐type (WT) littermates [33] in complementary contingent and non‐contingent models of fentanyl exposure. Translationally, *Reln*+/− mice more closely resemble the reduced RELN expression reported in several human neuropsychiatric disorders [16, 19, 20] rather than complete knockout of *Reln* expression. Using a multi‐phase fentanyl intravenous self‐administration (IVSA) paradigm, we examined acquisition, motivation, extinction, and cue‐induced fentanyl seeking to determine whether *Reln* haploinsufficiency alters opioid reinforcement or relapse‐like behavior. In parallel, we examined conditioned place preference (CPP), fentanyl‐induced locomotion, and Fos immunoreactivity in the dorsal striatum to assess opioid‐evoked neuronal activation under passive exposure to fentanyl. Altogether, this study dissociates the role of *Reln* in reinforcement processes from adaptive responding associated with fentanyl exposure.

## 2 Methods

### 2.1 Mice

All animal care and experimental procedures were approved by the UCSD Institutional Animal Care and Use Committee. Male and female *Reln*+/− and WT littermate controls were bred in house using the B6C3Fe a/a‐Relnrl/J line (The Jackson Laboratory, #000235) and maintained on a C57BL/6J background. Genotypes were determined by PCR‐based analysis of tail biopsies [28]. Mice were group‐housed (2–4 per cage), except during IVSA where mice were individualized immediately following catheterization, under a 12‐h light/dark cycle with ad libitum food and water except during behavioral testing where mice were maintained on a reverse light cycle. Mice were handled for a minimum of 3 days before the start of each experiment. For multi‐phase IVSA, *n* = 8 WT and *n* = 5 *Reln*+/− male mice, and *n* = 5 WT and *n* = 8 *Reln*+/− female mice were used across two cohorts. For naive home cage RT‐qPCR baseline control mice, *n* = 4 WT and *n* = 3 *Reln*+/− male mice, and *n* = 3 WT and *n* = 3 *Reln*+/− female mice were used. For locomotion during baseline and fentanyl or saline conditioning, *n* = 4 WT and *n* = 4 *Reln*+/− male, and *n* = 4 WT and *n* = 4 *Reln*+/− female mice were used per low (20 μg/kg) or high (80 μg/kg) dose. One mouse passed before test day and was just excluded for this day only, resulting in *n* = 4 WT and *n* = 3 *Reln*+/− male, and *n* = 4 WT and *n* = 4 *Reln*+/− for the low dose group. For Fos immunohistochemistry *n* = 4 WT and *n* = 3 *Reln*+/− male mice were used for the low dose and *n* = 3 WT and *n* = 3 *Reln*+/− for saline condition. Across all experiments, mice ranged from 12 to 16 weeks of age at the beginning of testing.

### 2.2 Drug Preparation

Powdered fentanyl citrate (Cayman Chemical Company) was dissolved in 0.9% saline to a stock concentration of 5 mg/mL. For IVSA, 5 mg/mL stock of fentanyl citrate and saline was further diluted to a working solution with saline for a final concentration of 0.025 mg/mL to allow a dynamic range of 15–1.5 μg/kg/infusion that was dependent on the duration of infusion and mouse weight. For acute exposure and CPP, 5 mg/mL stock of fentanyl citrate and saline was further diluted to a working solution with saline for a final concentration of 0.003 or 0.012 mg/mL to allow for 20 and 80 μg/kg IP injections, respectively.

### 2.3 Fentanyl IVSA

For IVSA procedures, mice were individually housed to prevent cage mates from damaging the back‐mounted catheters. Mice were food‐restricted to 80% of their normal intake to increase motivation for operant conditioning [34].

#### 2.3.1 Surgical Procedures

Mice underwent intravenous jugular catheterization using standard procedure [35, 36]. Following surgery, mice were given daily post‐operative care with intravenous flushes of heparinized saline containing cefazolin antibiotic to prevent clogging and infection. Once mice recovered to pre‐surgical body weight and had a dry wound area (3–5 days), the mice underwent fentanyl IVSA.

#### 2.3.2 Acquisition (FR1, 15 μg/kg/Infusion)

Self‐administration was conducted in standard operant‐conditioning chambers (Med Associates) equipped with two retractable levers positioned on one wall where an active lever was reinforced with intravenous delivery of fentanyl as opposed to an inactive lever that featured no consequences. At the start of each IVSA session, the white house lights were illuminated at the top of the arena, mice were given a passive infusion of fentanyl at the dose they would receive for the session to increase autoshaping of drug‐paired lever interaction [37], and a yellow availability light cue above the active lever was illuminated. Upon interaction with the active lever, the yellow availability light cue extinguished illumination, fentanyl was delivered, and a pure tone was played for 10 s. Active lever presses within this 10 s tone did not allow another infusion delivery. Following the 10 s tone, the availability light was illuminated again to signal another fentanyl infusion was available. Infusion numbers and active and inactive lever presses were recorded. The house lights were illuminated with both active and inactive levers extended for the entire session. Each session was 2 h in duration and conducted daily.

#### 2.3.3 De‐Escalating Dose–Response Testing

Following acquisition, mice underwent dose–response testing in which the fentanyl unit dose was sequentially reduced (15, 10, 5, and 1.5 μg/kg/infusion), with two consecutive 2‐h sessions at each dose under an FR1 schedule. Different unit doses (Supplemental Table 1) were delivered using RPM‐adjustable syringe pumps (Med Associates) loaded with a single fentanyl working solution (0.025 mg/mL). Infusion durations were predetermined for each unit dose, while pump RPM was adjusted daily according to each mouse's body weight to maintain the target dose. The corresponding RPM was calculated using the following equation:

$$\begin{array}{c} \text{weight adjusted}\;\mathrm{RPM}=\frac{\left(\text{Target dose}\left(\mathrm{mg}/\mathrm{kg}\right)\times \text{Body weight}\;\left(g\right)\right)/\left(\text{Fentanyl concentration}\;\left(\mathrm{mg}/\mathrm{mL}\right)\times \text{Infusion duration}\;\left(s\right)\right)}{\left(\text{pump constant}\;\left(0.195\right)\times \text{syringe cross}-\text{sectional area}\;\left(cm^{2}\right)\right)} \end{array}$$

Dose order was identical for all animals and dose–response sessions followed the same cue presentation pattern featured in acquisition. Mice were given an initial passive infusion of fentanyl at the start of the session using the dose they received for the session similar to acquisition. Infusion number, active and inactive lever presses, and total intake were recorded.

#### 2.3.4 Dose Reset Testing

Following dose–response testing, two “dose reset” sessions were conducted at 15 μg/kg/infusion dose to re‐stabilize responding to the acquisition dose.

#### 2.3.5 Progressive Ratio Testing

Following dose–response and dose‐reset sessions, motivation was assessed under a linear PR schedule conducted at the lowest unit dose (1.5 μg/kg/infusion). Under the PR schedule, the response requirement increased incrementally on a linear scale for each subsequent infusion (1, 2, 3, 4, …). The breakpoint was defined as the highest completed ratio resulting in infusion delivery. Active and inactive lever presses, infusions, and breakpoints were recorded. A putative outlier in breakpoint analysis was evaluated using simulation‐based residual testing (DHARMa R. package v.0.4.7) and it was not flagged for exclusion.

#### 2.3.6 Extinction and Cue‐Induced Fentanyl Seeking

Following PR testing, mice underwent extinction training in which lever presses had no programmed consequences (no fentanyl delivery or cues). Extinction sessions were 2 h and conducted daily for 10 sessions. A 30‐min cue‐induced fentanyl seeking session was conducted by reintroducing the previously fentanyl‐paired cues without drug delivery. Lever presses during cue‐induced fentanyl seeking were compared to the first 30 min of the final extinction session.

### 2.4 RT‐qPCR

Following cue‐induced fentanyl seeking, mice were euthanized immediately after the session termination. Naive mice were euthanized directly after being taken out of the homecage. Dorsal striatal tissue punches (+1.5–0.0 mm bregma) were collected and sectioned using a vibratome (Leica), flash frozen, and stored at −80°C. Total RNA was extracted from frozen tissues using Direct‐Zol RNA Miniprep (Zymo Research, #R2050). cDNA was synthesized from 100 ng RNA using the Maxima H Minus First Strand cDNA Synthesis Kit with dsDNase (Thermo Fisher Scientific, #K1682). RT‐qPCR was performed using the following primer sequences (Supplemental Table 2) and SsoAdvanced Universal SYBR Green Supermix (Bio‐Rad, #1725270). Gene expression was normalized to the housekeeping gene, *Gapdh*, using the 2^−ΔCt method [38].

### 2.5 Fentanyl CPP

CPP was conducted in a two‐compartment apparatus with distinct visual and tactile cues. On the pre‐test day, mice were allowed free access to both chambers for 30 min to assess baseline contextual preference. A counter‐biased design was used. If a mouse displayed over 55% preference for one chamber during the pre‐test session, the least preferred chamber was assigned as the fentanyl paired context. If no strong baseline preference was observed (≤ 55%), pairing assignment was counterbalanced across genotypes. Fentanyl conditioning consisted of eight total treatment days where treatments alternated daily (four fentanyl, four saline in opposite chambers). Mice were treated with fentanyl in two separate cohorts, receiving either a low (20 μg/kg) or a high dose (80 μg/kg). On saline‐pairing days, mice received an equivalent volume of saline as they would on fentanyl‐treated days and were confined to the alternate chamber. On the test day, mice were allowed free access to both compartments for 30 min in a drug‐free state.

All sessions were 30 min in length and recorded using red lighting and a camera positioned above the apparatus. CPP videos were analyzed using DeepLabCut v3.0 to track the estimated position of the mouse midpoint across frames using an 8‐pointed pose‐estimation framework. The mouse was scored as occupying a chamber when its centroid fell within that chamber's boundary. Time spent in each chamber was calculated as the number of frames assigned to each chamber divided by the video frame rate (30 frames per second). Videos were visually inspected to confirm accurate chamber assignments. The preference score was calculated as the change in percentage of time spent in the fentanyl‐paired chamber compared to the saline‐paired chamber between the pre‐test and test sessions.

### 2.6 Locomotion Analysis

Locomotor activity for baseline, fentanyl, or saline treatments was quantified using DeepLabCut v3 to track the mouse location across frames using 8‐pointed pose‐estimation. The total distance (meter) traveled was calculated as the total sum of the difference between the animal's centroid position in consecutive frames. The reported average speed (meter/min) was calculated by dividing the total distance by the total time in the session (30 min). All analyses were performed using custom Python and Matlab (Mathworks) scripts and were conducted blind to genotype.

### 2.7 Acute Fentanyl Exposure and Fos Immunostaining

In a separate cohort, mice received an acute fentanyl intraperitoneal (i.p.) injection (20 μg/kg, i.p.) and were perfused in 1× PBS followed by 4% PFA 90 min later. Brains were post‐fixed (overnight in 4°C, 4% PFA), cryoprotected (30% sucrose solution at 4°C, until sunk), and sectioned coronally using a microtome (Microm HM440E). Three mounted sections (20um) featuring the dorsal striatum (bregma 0.74 mm) per each genotype were used. Immunohistochemistry (IHC) for Fos protein was performed as follows: 1‐h incubation in blocking buffer [1× PBS (Millipore Sigma; # P4244‐100ML), 10% normal goat serum (Millipore Sigma; # S26‐M), 0.3% TritonX‐100 (Millipore Sigma; #X100‐5ML)] at room temperature followed by overnight incubation in Fos primary antibody dilution solution [1× PBS, 5% normal goat serum, 0.1% TritonX‐100, and 1:2500 anti‐Fos rabbit (Synaptic Systems; # 226008)] at 4°C, three 15‐min washes in washing buffer (1× PBS, 0.3% TritonX‐100), 2‐h incubation in secondary antibody solution [1× PBS, 5% normal goat serum, 0.1% TritonX‐100, and 1:500 Donkey anti‐Rabbit AlexaFluor‐680 (ThermoFisher; # A10043)] at room temperature, three 15‐min washes in washing buffer at room temperature, 30‐min incubation in 1× PBS with 600 nM DAPI at room temperature, two 15‐min washes in washing buffer at room temperature. Sections were coverslipped with mounting medium (Millipore Sigma, # 10981). Images of the dorsal striatum were acquired using a fluorescence microscope (Keyence; BZ‐X810) under identical exposure settings across animals. Cropped region‐of‐interest (ROI) images corresponding to the dorsal striatum were generated in ImageJ [39] using standardized anatomical boundaries of the corpus callosum, lateral ventricle, and anterior commissure. Automated cell quantification was performed using CellProfiler [40] to identify DAPI‐ and Fos‐positive nuclei. For each mouse, three coronal slices (six total sections including left and right hemispheres) were analyzed. The average number of Fos+/DAPI+ cells per section was calculated and used as the technical replicate for statistical analysis.

### 2.8 Statistical Analysis

All statistical analyses and data visualization were conducted in R (version 4.5.0). For IVSA, separate models were fitted for each experimental phase (acquisition, dose–response, dose reset, progressive ratio, extinction, and cue‐induced fentanyl seeking) where residuals were evaluated for model fitness and outliers using permutation‐based testing in R package DHARMa developed specifically for generalized linear mixed‐effects models (GLMMs) and linear mixed models (LMMs). IVSA count data (lever presses or infusions) were analyzed using negative‐binomially distributed GLMMs after failing to meet Gaussian assumptions while featuring overdispersion. IVSA continuous, positive data (total intake) were analyzed using gamma‐distributed GLMMs after failing to meet Gaussian assumptions while featuring overdispersion. For lever presses, infusions, or total intake responses, fixed effects included genotype, lever, day, session condition, and sex, with mice included as a random effect. For breakpoint response, fixed effects included genotype and sex. RT‐qPCR relative expression values were modeled with LMMs after meeting Gaussian assumptions to compare the fixed effects of genotype and sex for each gene of interest, where mice and their nested qPCR technical replicates were considered random effects. CPP data, which met Gaussian assumptions, were analyzed using a LMM to assess fixed effects of genotype, dose, sex, and change in preference for the fentanyl‐paired chamber, and mice as a random effect. Similarly, fentanyl‐induced locomotion (average speed) was analyzed using an LMM including genotype, dose, and sex as fixed effects. The percentage of Fos‐positive nuclei relative to total DAPI‐positive nuclei was modeled with a LMM to compare the fixed effect of genotype, where mice and their nested technical replicated slices were considered random effects. For GLMMs and LMMs, fixed effects and interactions were evaluated and reported for significance when *p* < 0.05 using Wald χ^2^ omnibus testing. Post hoc pairwise comparisons were performed using estimated means with Benjamini‐Hochberg false discovery rate (FDR) correction and reported for significance when pAdj < 0.05. We report significant interactions when post hoc comparison tests remain significant. Significant post hoc comparisons not involving genotype interactions are reported in the statistical tables but are not marked with asterisks in the figures. Exact test statistics and degrees of freedom are reported in the Supplemental Tables 3–6.

## 3 Results

### 3.1 *Reln* Haploinsufficiency Does Not Alter the Acquisition of Fentanyl Self‐Administration

To determine whether reduced *Reln* expression alters opioid reinforcement or relapse‐like responding, *Reln*+/− and WT littermates were assessed in a multi‐phase fentanyl IVSA paradigm, including acquisition, dose–response, dose reset, progressive ratio (PR), extinction, and a cue‐induced fentanyl seeking session (Figure 1A).

**FIGURE 1:**
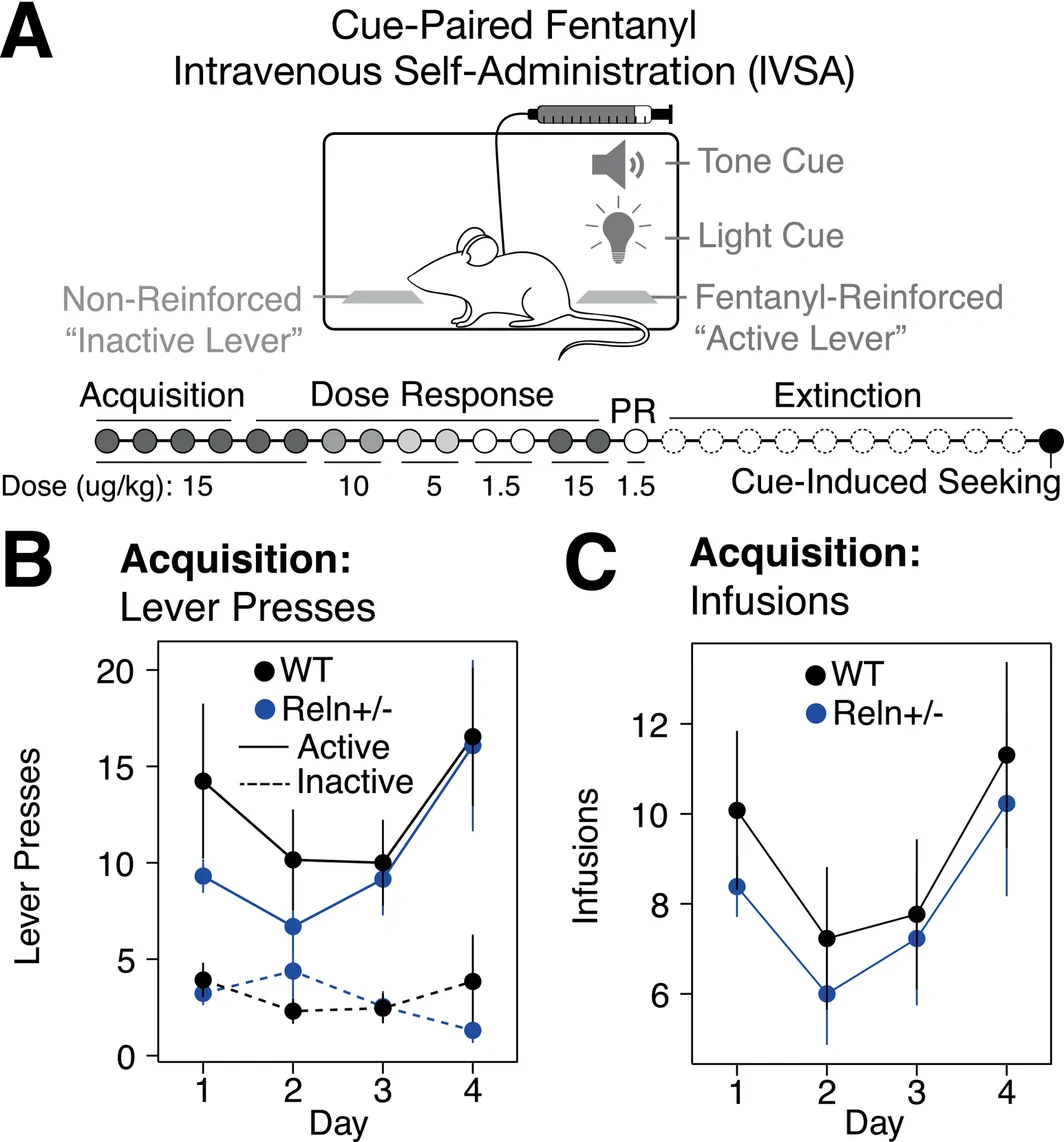
Assessing *Reln* haploinsufficiency within a fentanyl IVSA paradigm. (A) Schematic of fentanyl IVSA comparing Reln+/− (*n* = 13) and WT (*n* = 13) mice. B‐C Active (solid line) and inactive (dashed line) lever presses (B) or fentanyl infusions (C) across acquisition sessions (Days 1–4), collapsed across sex. Data are mean ± SEM.

During a four‐day acquisition phase (15 μg/kg/infusion), both genotypes acquired fentanyl self‐administration, as evidenced by a significant main effect of lever (χ^2^(1) = 10.82, *p* = 0.001, Supplemental Table 3.01), indicating that mice across both genotypes preferentially pressed the active over the inactive lever. This lever discrimination persisted across sessions, reflected in a significant lever × day interaction (χ^2^(3) = 9.911, *p* = 0.019, Supplemental Table 3.01). Importantly, there was no main effect of genotype on lever presses or infusions during acquisition (Figure 1B,C, Supplemental Tables 3.01 and 3.02), indicating that baseline fentanyl reinforcement learning was preserved in *Reln*+/− mice. Together, these data indicate that *Reln* haploinsufficiency does not modify baseline opioid reinforcement.

### 3.2 Reln Haploinsufficiency Does Not Alter Dose‐Dependent Fentanyl Responding

To determine whether *Reln* haploinsufficiency alters dose‐dependent fentanyl responding, we implemented a de‐escalating dose response phase in which the unit dose was reduced sequentially across sessions (15, 10, 5, 1.5 μg/kg/infusion). When modeled by dose, both genotypes exhibited the expected inverse relationship between unit dose and active lever presses, with responding increasing as the unit dose decreased (Figure 2A; lever × dose: χ^2^(3) = 14.51, *p* = 0.002; post hoc dose contrasts in Supplemental Table 3.03). Modeling sessions by individual day similarly confirmed the dose‐dependent pattern of lever responding (lever × day: χ^2^(7) = 20.17, *p* = 0.005; Supplemental Table 3.04). We detected a significant interaction between lever presses, genotype, and sex (χ^2^(1) = 6.222, *p* = 0.013; Supplemental Table 3.03). However, post hoc pairwise comparisons detected no significant genotype difference on either lever within either sex (Supplemental Figure 1A, Supplemental Table 3.03), indicating that this interaction does not reflect a direct genotypic effect on dose‐dependent responding.

**FIGURE 2:**
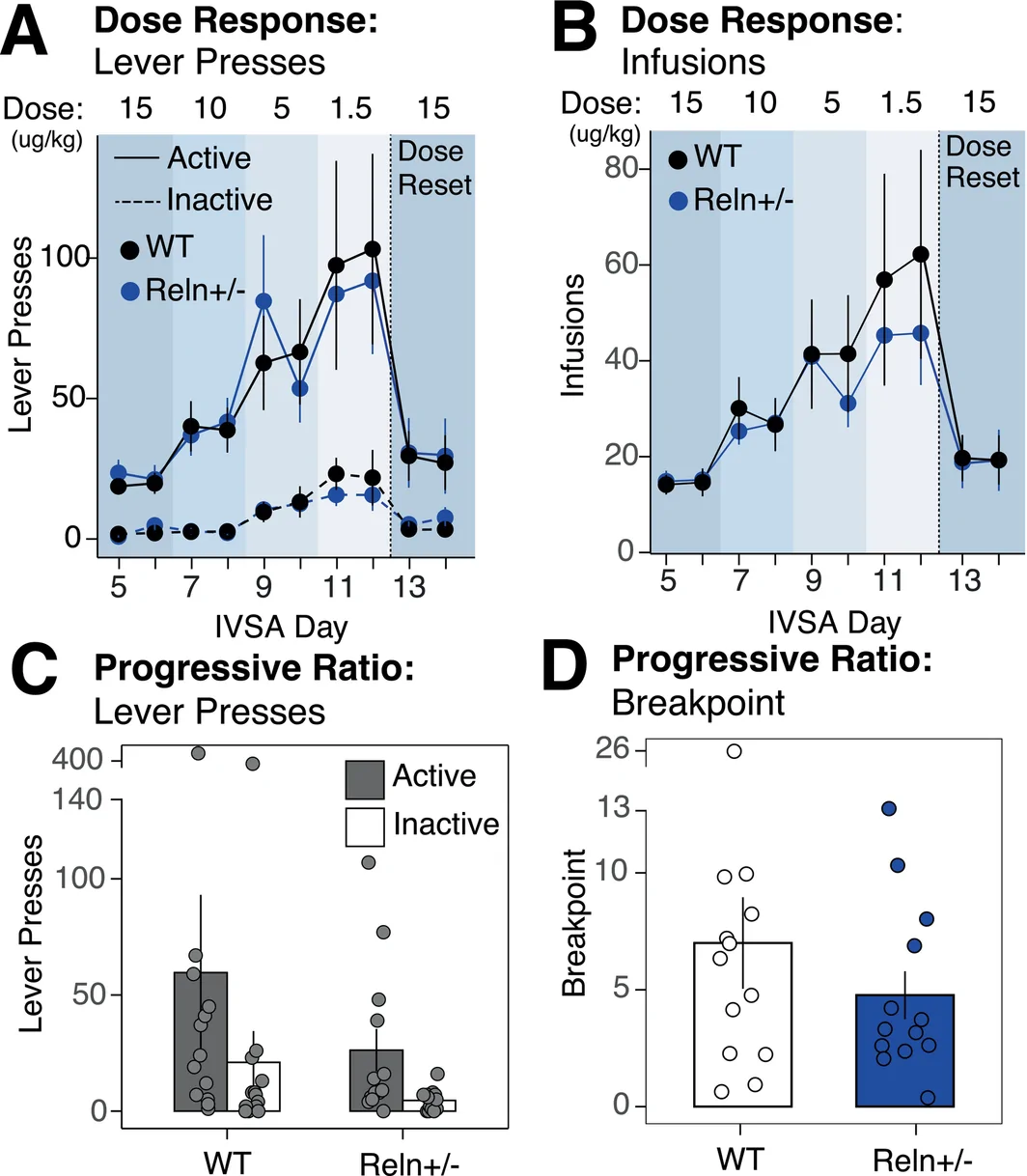
*Reln* haploinsufficiency does not alter fentanyl dose–response or motivation during intravenous self‐administration. A‐B Active (solid line) and inactive (dashed line) lever presses (A) or fentanyl infusions (B) across the dose–response phase, in which the unit dose was decreased sequentially from 15 to 10, 5, and 1.5 μg/kg/infusion over IVSA Days 5–12, followed by a dose reset to 15 μg/kg/infusion (Day 13–14; dotted vertical line). (C) Total active and inactive lever presses during the progressive ratio session, in which the response requirement increased with each successive infusion. (D) Breakpoint during the progressive ratio session, aggregated across sex. Sex‐stratified breakpoint data are shown in Supplemental Figure 2B. Data are mean ± SEM. (WT, *n* = 13; Reln+/−, *n* = 13).

Fentanyl infusions followed a comparable pattern, increasing as unit dose decreased when modeled by dose (Figure 2B; main effect of dose: χ^2^(3) = 36.14, *p* < 0.001, Supplemental Table 3.05) or by session day (main effect of day: χ^2^(7) = 90.52, *p* < 0.001, Supplemental Table 3.06), with no genotype effects detected in either model. Total fentanyl intake similarly varied as a function of dose (main effect of dose: χ^2^(3) = 39.62, *p* < 0.001, Supplemental Table 3.07) and session day (main effect of day: χ^2^(7) = 146.4, *p* < 0.001, Supplemental Table 3.08), producing an inverted U‐shaped function when collapsed across doses (Supplemental Figure 1C–E), again with no genotype differences.

Following dose–response testing, mice were returned to the acquisition dose (15 μg/kg/infusion) for two dose reset sessions. Both genotypes maintained lever discrimination during this phase (Figure 2A; main effect of lever: χ^2^(1) = 17.12, *p* < 0.001, Supplemental Table 3.09), with no change in responding across the sessions and no genotype differences in lever presses or infusions (Supplemental Tables 3.09 and 3.10), indicating stable performance at the original acquisition dose.

Together, these findings demonstrate that both genotypes responded appropriately to changes in unit dose and that *Reln* haploinsufficiency did not produce a consistent effect on dose‐dependent fentanyl responding.

### 3.3 *Reln* Haploinsufficiency Is Associated With a Sex‐Dependent Difference in Progressive Ratio Breakpoint

Following the two dose‐reset sessions, motivation for fentanyl was assessed under a linear PR schedule conducted at the lowest dose in the dose–response (1.5 ug/kg/infusion). Mice showed strong lever bias overall (Figure 2C; main effect of lever: χ^2^(1) = 6.964, *p* = 0.008; Supplemental Table 3.11), with no detectable genotype differences in lever presses (Supplemental Table 3.11). Breakpoint showed no main effect of genotype (χ^2^(1) = 0.391, *p* = 0.532) or sex (χ^2^(1) = 0.391, *p* = 0.532), but a significant genotype × sex interaction was detected (Figure 2D; χ^2^(1) = 4.402, *p* = 0.036; Supplemental Table 3.12). This interaction reflects a single post hoc contrast that is significant whether expressed as a sex difference within WT mice (WT males>WT females) or as a genotype difference within males (WT>Reln+/−); both descriptions refer to the same single contrast (Supplemental Figure 2B; estimate = 0.48, *z* = −2.365, adjP = 0.018; Supplemental Table 3.12). No significant difference was detected between female and male *Reln*+/− mice or between genotypes in females. In summary, PR breakpoint did not differ by genotype overall, and the genotype × sex interaction reflects a reduced breakpoint in male *Reln*+/− mice relative to WT males.

### 3.4 *Reln* Haploinsufficiency Does Not Alter Extinction or Cue‐Induced Fentanyl Seeking

During extinction, no significant effect of lever (χ^2^(1) = 0.532, *p* = 0.466), genotype (χ^2^(1) = 0.201, *p* = 0.654), or day (χ^2^(9) = 15.969, *p* = 0.068) was detected (Figure 3A; Supplemental Table 3.13), indicating that lever discrimination was lost in the absence of cue presentation and fentanyl infusions in both WT and *Reln*+/− mice, but overall response rates did not decline across sessions. It should be noted that mice did not reduce lever pressing as expected from classic extinction models; rather, the active‐versus‐inactive lever discrimination present throughout acquisition was lost once cues and fentanyl were withheld, consistent with persistent, non‐discriminating lever pressing under extinction conditions.

**FIGURE 3:**
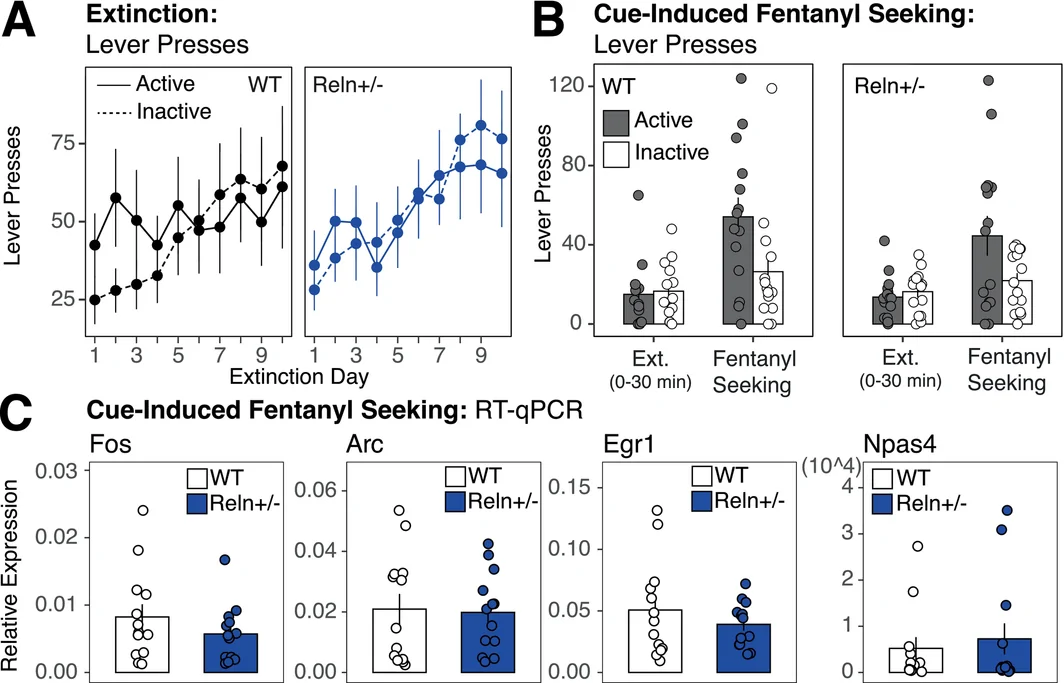
*Reln* haploinsufficiency does not alter extinction, cue‐induced fentanyl seeking, and dorsal striatal immediate early genes expression during cue‐induced fentanyl seeking. (A) Active (solid line) and inactive (dashed line) lever presses across 10 daily extinction sessions for WT (black) and Reln+/− (blue) mice. (B) Comparison of active and inactive lever presses during the final 30 min of the last extinction session (Ext.) and during the 30‐min cue‐induced fentanyl seeking session, aggregated across sex. Sex‐stratified data are shown in Supplemental Figure 3B. (C) Relative expression of immediate early genes *Fos, Arc, Egr1, and Npas4* in the dorsal striatum following cue‐induced fentanyl seeking, normalized to *Gapdh*, in WT and *Reln*+/− mice. Data are mean ± SEM (WT, *n* = 13; Reln+/−, *n* = 13).

During the cue‐induced fentanyl seeking session, in the presence of fentanyl‐associated cues without fentanyl delivery, both genotypes exhibited robust cue‐induced fentanyl seeking displayed by increased active lever responding compared to the last extinction session, demonstrated by a significant main effect of session condition (Figure 3B; χ^2^(1) = 14.43, *p* < 0.0001; Supplemental Table 3.14). Post hoc comparisons confirmed that both genotypes and both sexes increased responding from extinction to the cue‐induced fentanyl seeking session (all adjP < 0.001; Supplemental Table 3.14). Three further interactions were detected: session condition × genotype (χ^2^(1) = 4.082, *p* = 0.043), session condition × sex (χ^2^(1) = 5.028, *p* = 0.025), and session condition × genotype × sex (χ^2^(1) = 5.660, *p* = 0.017; Supplemental Table 3.14). Post hoc analysis identified a single significant contrast: WT females showed greater active lever pressing than WT males during the cue‐induced fentanyl seeking session (Supplemental Figure 3B; estimate = 2.358, *z* = 2.023, adj*P* = 0.043). Critically, no significant genotype difference was detected within either sex under either session condition, indicating that this is a sex difference within WT animals rather than a genotype‐specific effect.

To further support that mice exhibit a cue‐induced fentanyl seeking phenotype considering the lack of classic extinction, we compared cumulative lever presses during the two‐hour extinction sessions with the 30‐min cue‐induced fentanyl seeking session (Supplemental Figure 3C,D). Active lever discrimination was present from the start of the cue‐induced fentanyl seeking session and absent during the first 30 min of the final extinction session. This analysis does not establish reinstatement in the classical sense, but indicates that responding during the cue‐reinstated seeking session was cue‐directed in a manner that differs from extinction.

Consistent with the absence of genotypic differences in cue‐induced fentanyl seeking behavior, the expression of IEGs (*Fos, Arc, Egr1, Npas4*) in the dorsal striatum did not differ between genotypes or sexes following the cue‐induced fentanyl seeking session (Figure 3C; Supplemental Table 4.01), nor in an independent naive home‐cage cohort (Supplemental Figure 4A,C; Supplemental Table 4.02), indicating no detectable baseline genotype difference in striatal IEG expression.

Together, these findings indicate that *Reln* haploinsufficiency does not alter cue‐induced fentanyl seeking in either sex. The sex difference in seeking magnitude was observed within WT mice only, and given the subgroup sizes we regard it as exploratory rather than as evidence for a genotype‐specific sex effect.

### 3.5 *Reln* Haploinsufficiency Enhances Non‐Contingent Acute Fentanyl‐Induced Locomotion and Dorsal Striatal *Fos* Expression Without an Effect on CPP

To examine opioid‐evoked responses independent of instrumental contingencies, we evaluated passive fentanyl exposure using CPP, locomotor analysis, and Fos immunoreactivity in the dorsal striatum as a marker of neuronal activation.

CPP doses (20 or 80 μg/kg, i.p.) were selected to approximate contingent intake observed during IVSA at the lowest and highest unit doses, respectively (Figure 4A). Analysis of absolute chamber occupancy detected a session x chamber interaction (Figure 4B; χ^2^(1) = 70.297, *p* < 0.001; Supplemental Figure 5A,B, Supplemental Table 5.01), confirming that mice acquired preference for the fentanyl paired chamber. Additional interactions were detected between genotype, dose, and chamber (χ^2^(1) = 4.790 *p* = 0.029), between genotype, session, and chamber (χ^2^(1) = 4.148, *p* = 0.042), and between sex and chamber (χ^2^(1) = 7.077, *p* = 0.008). Post hoc comparisons of test‐day chamber discrimination reached significance in *Reln*+/− males at the low dose (estimate = 0.768, z = −3.027, adj*P* = 0.002) and in WT males at the high dose (estimate = 0.846, z = −2.214, adj*P* = 0.027). We emphasize that these are within‐genotype contrasts of chamber occupancy, not comparisons between genotypes; no between‐genotype contrast reached significance at either dose in either sex (Supplemental Table 5.01). Consistent with this, the change in preference score for the fentanyl‐paired chamber did not differ by genotype, dose, or sex (Figure 4C; Supplemental Table 5.02).

**FIGURE 4:**
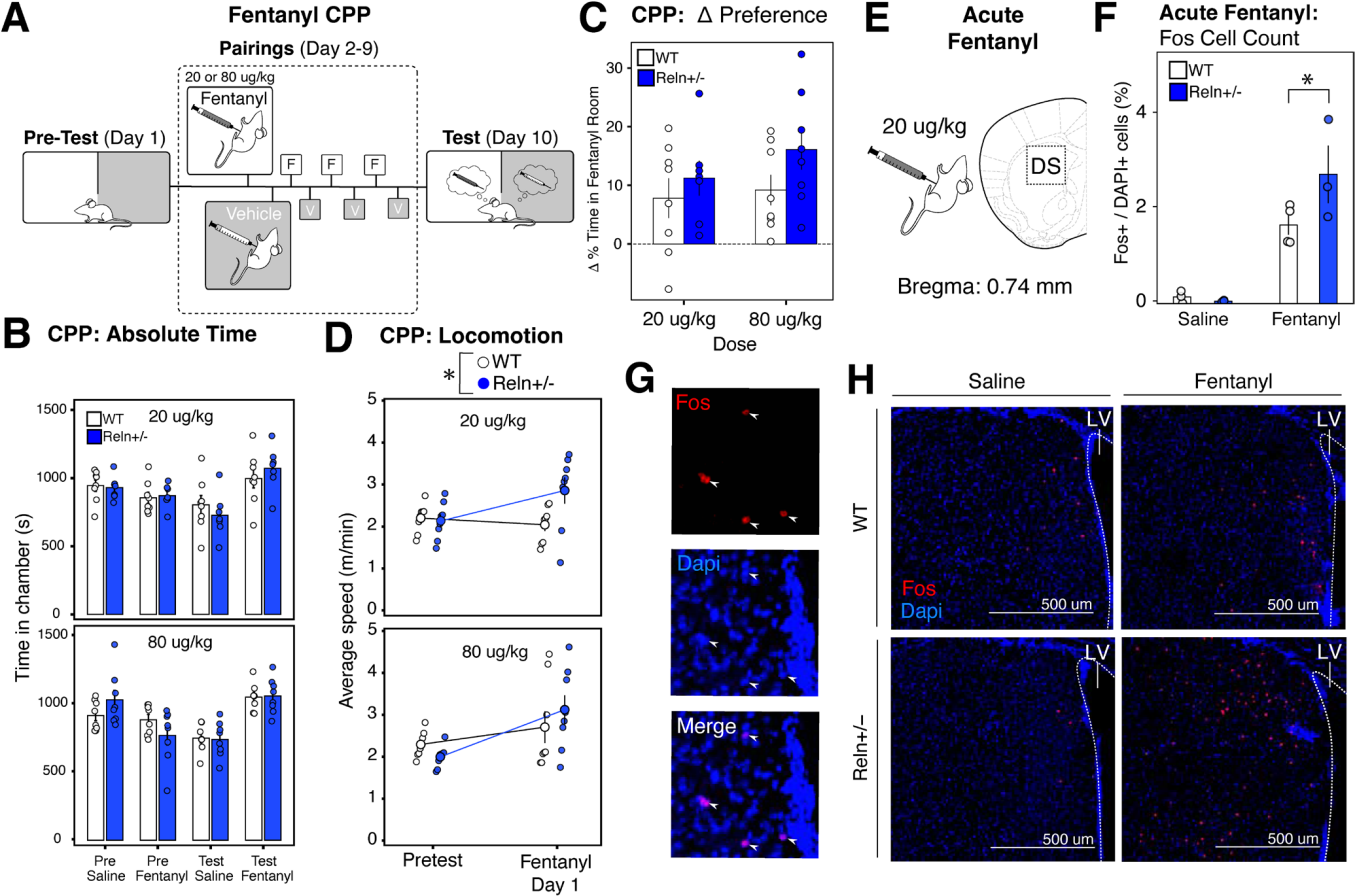
*Reln* haploinsufficiency enhances acute fentanyl‐induced locomotion and dorsal striatal Fos immunoreactivity without altering conditioned place preference. (A) Schematic of the CPP paradigm. (B) Absolute time spent in saline or fentanyl paired chamber by 20 ug/kg (top) or 80 ug/kg (bottom) dose. (C) Change in percent time spent in the fentanyl‐paired room at 20 μg/kg (left) or 80 μg/kg (right) doses. (D) Average locomotor speed (total distance/total time; meter/min) during the pretest or first CPP conditioning session following acute fentanyl injection at 20 μg/kg (top) or 80 μg/kg or (bottom), aggregated across sex. Sex‐stratified locomotion is shown in Supplemental Figure 5. Data are mean ± SEM (20 ug/kg—female: WT *n* = 4, Reln+/− *n* = 4; male: WT *n* = 4, Reln+/− *n* = 3; 80 ug/kg—WT *n* = 4, Reln+/− *n* = 4; male: WT *n* = 4, Reln+/− *n* = 4;). (E‐F) Region of interest (E) for quantification of Fos + cells as a proportion of total DAPI+ cells in the dorsal striatum (F) of a different cohort of male mice following acute fentanyl (20 μg/kg, i.p.) or saline injection. (G) Representation for Fos+/DAPI+ colabeled cell for Fos + cell quantification. (H) Representative fluorescent image of the dorsal striatum from WT (top) and *Reln*+/− (bottom) male mice following acute injection of fentanyl (20 μg/kg; right) or saline (left). Fos immunoreactivity is shown in red; DAPI nuclear counterstain is in blue. Scale bar, 500 μm. Data are mean ± SEM. Fentanyl: WT, *n* = 4; *Reln*+/−, *n* = 3 mice; Saline: WT, *n* = 3; *Reln*+/−, *n* = 3 mice. *p < 0.05.

Locomotor activity was quantified across fentanyl conditioning sessions. Comparing the drug‐free baseline (pretest) session with the first fentanyl conditioning session revealed a main effect of treatment (χ^2^(1) = 20.852, *p* < 0.001) and, critically, a genotype × treatment interaction (χ^2^(1) = 15.925, *p* < 0.001; Figure 4D; Supplemental Table 5.03). Post hoc comparisons showed that WT and *Reln*+/− mice did not differ at baseline, but *Reln*+/− mice showed higher rates of fentanyl‐induced locomotion than WT mice (estimate = 0.794, z = −3.047, adj*P* = 0.0023). A treatment × dose × sex interaction was also detected (χ^2^(1) = 4.242, *p* = 0.039), where an overall sex difference was found between females and males at the lower 20 μg/kg dose (estimate = 0.6729, z = −3.3408, *p* = 0.0008). Indeed, males show increased fentanyl‐induced locomotion for both doses (low: estimate = 0.7045, z = −3.5405, adjP < 0.001; high: estimate = 0.6882, z = −3.778, adj*P* < 0.001) where females do not significantly exhibit fentanyl‐induced hyperlocomotion relative to the baseline. Within females, locomotion was higher following 80 μg/kg dose than the 20 μg/kg dose, suggesting a female‐specific dose‐dependent effect on locomotion following acute fentanyl exposure (estimate = 0.756, *z* = −2.3615, *p* = 0.018). Averaged across all conditioning days, the genotype × treatment interaction remained significant (χ^2^(1) = 4.418, *p* = 0.036; Supplemental Table 5.04). This interaction is produced by *Reln*+/− mice showing increased hyperlocomotion following fentanyl whereas WT did not (estimate = 1.2284, *z* = 3.8822, *p* = 0.0001). Importantly, saline‐session locomotion did not differ by genotype (Supplemental Table 5.03), confirming that the genotype difference is specific to the acute locomotor response to fentanyl rather than a pre‐existing locomotor phenotype.

To determine whether this behavioral difference was accompanied by altered neuronal activation, a separate cohort of male mice received an acute fentanyl injection at the low dose (20 μg/kg, i.p.) or saline, and Fos immunoreactivity was quantified 90 min later in the dorsal striatum (Figure 4E–H). The low dose was selected because it produced the larger genotype separation in locomotor activation, and the dorsal striatum was selected because prior work from our laboratory identified altered dorsal striatal Fos activation in *Reln*+/− mice following cocaine exposure [28]. Quantifying Fos‐positive (Fos+) cells as a percentage of total DAPI‐positive nuclei, we detected a main effect of treatment (χ^2^(1) = 68.288, *p* < 0.001), a main effect of genotype (χ^2^(1) = 4.204, *p* = 0.040), and a treatment × genotype interaction (χ^2^(1) = 5.899, *p* = 0.015; Figure 4F; Supplemental Table 6). Fentanyl increased Fos immunoreactivity in both genotypes (WT: estimate = −1.480, *z* = −3.553, adj*P* = 0.002; *Reln*+/−: estimate = −2.712, z = −6.091, adj*P* < 0.001). Critically, the genotypes did not differ under saline (estimate = 0.096, z = 0.216, adj*P* = 0.832) but did differ following fentanyl, with *Reln*+/− mice showing greater Fos immunoreactivity than WT mice (estimate = −1.136, z = −2.728, adj*P* = 0.013).

Together, these findings demonstrate that overall *Reln* haploinsufficiency enhances acute fentanyl‐induced locomotion and neuronal activation in the dorsal striatum, without a corresponding change in CPP.

## 4 Discussion

This study provides the first characterization of *Reln* in the context of opioid‐related behaviors. Using a multi‐phase fentanyl IVSA paradigm, we found that *Reln* haploinsufficiency did not alter acquisition, extinction, or cue‐induced fentanyl seeking. Progressive ratio testing detected a genotype x sex interaction on breakpoint, driven by an elevated breakpoint in WT males, which we regard as provisional given the subgroup sizes. In complementary non‐contingent assays, the most prominent effect of reduced *Reln* expression emerged under conditions of acute pharmacological challenge where *Reln*+/− mice exhibited enhanced fentanyl‐induced locomotion and elevated Fos immunoreactivity in the dorsal striatum.

### 4.1 *Reln*‐Dependent Modulation of Acute Fentanyl‐Induced Locomotor and Striatal Activation

*Reln*+/− mice exhibited enhanced fentanyl‐induced locomotion relative to their own drug‐free baseline and relative to WT mice, together with increased dorsal striatal Fos immunoreactivity following acute exposure at the lower dose of fentanyl (20 μg/kg). This locomotor difference was most evident upon the first fentanyl exposure and diminished across repeated conditioning days, and at these doses the fentanyl‐induced increase over baseline was carried by males; we therefore interpret it as an acute response to initial fentanyl exposure at behaviorally relevant doses rather than a sustained hyperlocomotor phenotype. In both cases the genotypes were indistinguishable in the absence of fentanyl, indicating that these are drug‐induced rather than constitutive differences. No genotypic difference in *Fos* expression was detected in the dorsal striatum following cue‐induced fentanyl seeking, although the absence of a procedure‐matched drug‐free control means a floor effect cannot be excluded. These findings, together with the absence of changes during cue‐induced fentanyl seeking or CPP, suggest that *Reln* preferentially modulates acute pharmacological activation in the striatum rather than learned cue‐driven neuronal activation.

These findings extend prior work linking Reelin signaling to drug‐induced neuronal activation. Notably, similar phenotypes have been reported in psychostimulant models. De Guglielmo et al. [28] demonstrated that *Reln* haploinsufficient mice exhibit enhanced cocaine‐induced hyperlocomotion accompanied by increased *Fos* expression in the dorsal striatum, without a significant alteration in cocaine CPP. This parallel across two pharmacologically distinct drug classes suggests that *Reln* has a specific role in modulating drug‐induced responses, separate from the learned associations that drive cue‐induced drug seeking. Consistently, fentanyl has been shown to recruit distinct dorsal striatal ensembles and to drive lasting striatal circuit adaptations [41].

### 4.2 *Reln* Haploinsufficiency Does Not Alter Opioid Reinforcement

During fentanyl IVSA, *Reln*+/− mice exhibited unaltered acquisition, maintenance, and extinction of fentanyl self‐administration, and total fentanyl intake did not differ by genotype during dose–response testing. Fentanyl CPP was similarly unaffected. Together, these results indicate that *Reln* haploinsufficiency does not robustly alter the reinforcing effects of fentanyl or the expression of learned drug‐seeking behavior.

The sex‐related observations across the IVSA phases warrant explicit consideration together. Detection of a genotype × sex interaction for breakpoint during the progressive ratio assay reflected a reduced breakpoint in male Reln+/− mice relative to WT males, alongside higher breakpoints in WT males than WT females. Although significant lever × genotype and lever × genotype × sex interactions emerged during dose–response testing, and a session × condition × genotype interaction emerged during cue‐induced fentanyl seeking, in no case did genotypes differ significantly within a given sex or session condition after post hoc correction. Moreover, the insufficient power for our sex‐specific subgroup sizes does not support the interpretation of these findings with full certainty. Accordingly, both observations should be considered as provisional and serve as motivation for a replication study in an adequately powered, prospectively designed cohort rather than as evidence for a sex‐dependent role of Reelin signaling in motivation.

Existing literature supports that *Reln*‐dependent effects on drug‐induced behavior are not uniform across drug classes, dose, and experimental approaches. Methamphetamine‐induced hyperlocomotion was attenuated, rather than enhanced, in *Reln* haploinsufficient mice in a dose‐dependent manner [29], a direction opposite to the fentanyl‐induced locomotion observed here. *Reln* knockdown specifically in the nucleus accumbens reduced cocaine CPP without altering cocaine‐induced locomotion [27], a dissociation that contrasts with the present findings of enhanced fentanyl locomotion in the absence of altered CPP. This divergence may also reflect differences in developmental stages and regional specificity of *Reln* manipulation. The present study used germline *Reln* haploinsufficient mice, in which reduced *Reln* expression occurs throughout development and across all brain regions. In contrast, prior cocaine IVSA experiments used adult, region‐specific knockdown confined to the nucleus accumbens of rats [27], making direct comparisons difficult.

Collectively, these findings indicate that the influence of Reelin signaling on drug‐evoked behavior depends on the pharmacological mechanism of the drug, the brain region, and cell type in which Reln is manipulated, and the developmental timing of the manipulation.

## 5 Study Limitations

Several limitations of the present study warrant consideration. First, the use of germline *Reln* haploinsufficient mice precludes dissociation of developmental versus adult roles of Reelin signaling. Given the critical role of Reelin signaling in neuronal migration, dendritic maturation, and synaptogenesis during development [7, 33, 42], it is possible that the locomotor and striatal‐activation phenotypes reported here reflect developmental adaptations rather than an acute requirement for Reelin signaling in the adult brain. This interpretation is further complicated by the system‐wide nature of the haploinsufficiency where reduced *Reln* expression occurs across all brain regions and cell types throughout the lifespan, making it impossible to attribute the observed effects specifically to dorsal striatal Reelin signaling. Future studies using inducible time‐, circuit‐ and cell‐type‐specific manipulations of *Reln* expression in adolescent or adult animals will be required to further isolate the contribution of Reelin signaling in the dorsal striatum to opioid‐induced locomotion and neuronal activation.

Second, the Fos immunoreactivity results, while statistically significant, were obtained in males only; whether the enhanced fentanyl‐induced Fos response generalizes to females remains to be determined. An independent replication in a larger, mixed‐sex cohort will be necessary to confirm the robustness of this finding and to determine whether the enhanced Fos response generalizes across sexes.

Third, molecular analyses were restricted to a small panel of IEGs at a single timepoint, which do not capture all IEGs [43] nor the full transcriptional or epigenetic landscape of opioid exposure [44, 45]. Although we did not detect a baseline difference in IEG between genotypes, the study lacks a saline‐matched cohort to fully interpret IEG expression and should be interpreted with this caveat in consideration. In addition, the absence of a genotypic difference in *Fos* expression during cue‐induced fentanyl seeking or at baseline in naive mice was assessed in a single brain region at a single timepoint; it remains possible that *Reln*‐dependent differences in neuronal activation emerge in other brain regions, in other cell populations, or at different phases of the IVSA protocol.

Finally, although the present study detected significant genotype × sex interactions during both PR testing and cue‐induced fentanyl seeking, the study was not specifically designed or powered to fully characterize sex as a biological variable. The subgroup sample sizes were modest (males: *n* = 8 WT, *n* = 5 *Reln*+/−; females: *n* = 5 WT, *n* = 8 *Reln*+/−; CPP: *n* = 4 per genotype × sex × dose), which limits the reliability and interpretability of these sex‐stratified effects and does not support dose‐ or sex‐resolved conclusions from the CPP dataset. Future studies specifically designed and powered to test sex as a biological variable will be necessary to determine whether *Reln*‐dependent modulation of opioid reinforcement differs between males and females.

## 6 Conclusions

Collectively, these findings establish that Reelin preferentially modulates the acute pharmacological effects of opioids within the dorsal striatum rather than directing the associative learning processes that drive opioid reinforcement and drug‐seeking. This dissociation positions *Reln+/− mice* as a valuable model for studying how Reelin signaling shapes neuronal responses to opioids, with implications for understanding the molecular mechanisms underlying opioid sensitivity.

## Funding

This work was supported by NIH R01DA056602 to F.T.; and NIGMS T32 Training Grant (2T32GM133351) and 1F31DA063338‐01A1 to S.D.

## Conflicts of Interest

The authors declare no conflicts of interest.

## Supporting information


**Supplemental Figure 1** Sex‐stratified behavioral responses across fentanyl IVSA phases in WT and *Reln*+/− mice. (A, B) Active (solid line) and inactive (dashed line) lever presses (A) or infusions (B) across all IVSA sessions (Day 1–15), stratified by sex and genotype. (C–E) Total fentanyl intake (ug) averaged across the two sessions at each unit dose during the dose response phase for WT (*n* = 13, black) and *Reln*+/− (*n* = 13, blue) mice (C) or separated by females (D; WT *n* = 5, Reln+/− *n* = 8) and males (E; WT *n* = 8, Reln+/− *n* = 5). Data are mean ± SEM.
**Supplemental Figure 2**. Sex‐stratified progressive ratio lever pressing and breakpoint. (A‐B) Total lever presses stratified by sex (A) or breakpoint stratified by sex (B) during the progressive ratio session. Data are faceted by genotype and sex (female: WT *n* = 5, Reln+/− *n* = 8; male: WT *n* = 8, Reln+/− *n* = 5). Data are mean ± SEM. *p < 0.05.
**Supplemental Figure 3**. Cumulative lever presses for IVSA phases and sex‐stratified behavioral and molecular responses across fentanyl extinction and cue‐induced fentanyl seeking in WT and *Reln*+/− mice. (A) Active (solid line) and inactive (dashed line) lever presses during extinction phase (Extinction, Day 1–10), faceted by sex and genotype. (B) Comparison of active and inactive lever presses during the first 30 min of the last extinction session (Ext., Day 10) and during the subsequent 30 min cue‐induced fentanyl seeking session, faceted by genotype and sex. (C‐D) Cumulative summation of lever presses per each IVSA phase (C), stratified by sex (D). In (C) and (D), active lever presses are shown as solid lines and inactive lever presses as dashed lines. Data are faceted by genotype and/or sex (female: WT *n* = 5, Reln+/− *n* = 8; male: WT *n* = 8, Reln+/− *n* = 5). Data are mean ± SEM. *p < 0.05.
**Supplemental Figure 4**. Sex‐stratified IEG RT‐qPCR for naive and cue‐induced fentanyl seeking in WT and *Reln*+/− mice. (A) Relative expression (2^−ΔCt) of *Fos, Arc, Egr1*, and *Npas4* expression in the dorsal striatum from naive mice from the homecage, normalized to Gapdh; faceted by genotype. (B) Relative expression (2^−ΔCt) of *Fos, Arc, Egr1*, and *Npas4* expression in the dorsal striatum following cue‐induced fentanyl seeking, normalized to Gapdh; faceted by sex and genotype. (C) Relative expression (2^−ΔCt) of *Fos, Arc, Egr1*, and *Npas4* expression in the dorsal striatum from naive mice from the homecage, normalized to Gapdh; faceted by sex and genotype. Data are mean ± SEM (cue‐induced fentanyl seeking: Female: WT *n* = 5, *Reln*+/− *n* = 8; Male: WT *n* = 8, *Reln*+/− *n* = 5—Naive: Female: WT *n* = 3, *Reln*+/− *n* = 3; Male: WT *n* = 4, *Reln*+/− *n* = 3).
**Supplemental Figure 5**. Daily locomotion during CPP conditioning and sex‐stratified fentanyl CPP analysis. (A) Absolute time spent (s, seconds) per saline‐ or fentanyl‐paired room for each dose stratified by genotype and sex. (B) Change in percent time spent in the fentanyl‐paired room on the test day relative to baseline, for mice conditioned at 20 μg/kg or 80 μg/kg, faceted by sex and genotype. (C) Average locomotor speed (total distance/total time) during the pretest or first CPP conditioning session following acute fentanyl injection at 20 or 80 μg/kg, faceted by genotype and sex. (D) Average locomotor speed (total distance/total time) for acute fentanyl injection at 20 or 80 μg/kg or saline control when all days per treatment all averaged, faceted by genotype. (E, F) Locomotion for pretest or fentanyl/saline CPP conditioning days across all days faceted by genotype (E) and sex (F). Data are mean ± SEM. Locomotion, baseline, and conditioning: Female—WT *n* = 4; Reln+/− *n* = 4; Male—WT *n* = 4, Reln+/− *n* = 4 per each dose. Test Day: Female—WT *n* = 4; Reln+/− *n* = 4; Male—WT *n* = 4, Reln+/− *n* = 3 for low dose and WT *n* = 4; Reln+/− *n* = 4; Male—WT *n* = 4, Reln+/− *n* = 4 for high dose.

**Supplementary Table 1** Fentanyl unit‐dose delivery parameters for intravenous self‐administration.
**Supplementary Table 2**. Primer sequences used for RT‐qPCR. Forward and reverse primer sequences (5′ → 3′) for each target immediate early gene (*Fos, Arc, Egr1, Npas4*) and the *Gapdh* housekeeping gene used for normalization.
**Supplementary Table 3.01–3.14**. Statistical results for fentanyl IVSA behavior. Outputs for each IVSA phase (acquisition, dose–response, dose reset, progressive ratio, extinction, and cue‐induced fentanyl seeking) modeling lever presses, infusions, intake, and breakpoint.
**Supplementary Table 4.01–4.02**. Statistical results for dorsal striatal RT‐qPCR. Linear‐model outputs for immediate early gene expression (*Fos, Arc, Egr1, Npas4*) following cue‐induced fentanyl seeking (4.01) and in naïve mice (4.02), with genotype and sex as fixed effects and post hoc estimated marginal means comparisons.
**Supplementary Table 5.01–5.04**. Statistical results for conditioned place preference and fentanyl‐induced locomotion. Outputs for absolute chamber occupancy (5.01), Δ% preference (5.02), and fentanyl‐induced locomotion, comparing baseline versus fentanyl conditioning day 1 (5.03) and saline versus fentanyl across conditioning days (5.04), each with genotype, dose, treatment, and/or sex as fixed effects and post hoc estimated marginal means comparisons.
**Supplementary Table 6**. Statistical results for dorsal striatal Fos immunoreactivity. Linear mixed model output for the percentage of Fos+/DAPI+ cells, with genotype as a fixed effect, mice and nested slices as random effects, and post hoc estimated marginal means comparisons.

## Data Availability

The data that support the findings of this study are available from the corresponding author upon reasonable request.

## References

[1] L. Degenhardt , J. Grebely , J. Stone , et al., “Global Patterns of Opioid Use and Dependence: Population Harms, Interventions, and Future Action,” Lancet 394, no. 10208 (2019): 1560. 10.1016/S0140-6736(19)32229-9 PMC7068135 31657732

[2] Y. I. Hser , V. Hoffman , C. E. Grella , and M. D. Anglin , “A 33‐Year Follow‐Up of Narcotics Addicts,” Archives of General Psychiatry 58, no. 5 (2001): 503–508. 10.1001/archpsyc.58.5.503 11343531

[3] B. J. Everitt and T. W. Robbins , “Neural Systems of Reinforcement for Drug Addiction: From Actions to Habits to Compulsion,” Nature Neuroscience 8, no. 11 (2005): 1481–1489. 10.1038/nn1579 16251991

[4] J. Cucinello‐Ragland , T. Lintz , and J. Moron , “Neurobiology of Opioid Use Disorder,” in New Opioid Receptor Modulators and Agonists (Academic Press, 2026).

[5] V. Bisagno and J. L. Cadet , “Expression of Immediate Early Genes in Brain Reward Circuitries: Differential Regulation by Psychostimulant and Opioid Drugs,” Neurochemistry International 124 (2019): 10–18. 10.1016/j.neuint.2018.12.004 30557593

[6] G. D'Arcangelo , K. Nakajima , T. Miyata , M. Ogawa , K. Mikoshiba , and T. Curran , “Reelin Is a Secreted Glycoprotein Recognized by the CR‐50 Monoclonal Antibody,” Journal of Neuroscience 17, no. 1 (1997): 23–31. 10.1523/JNEUROSCI.17-01-00023.1997 PMC6793694 8987733

[7] Y. Ikeda and T. Terashima , “Expression of Reelin, the Gene Responsible for the Reeler Mutation, in Embryonic Development and Adulthood in the Mouse,” Developmental Dynamics 210, no. 2 (1997): 157–172. 10.1002/(SICI)1097-0177(199710)210:2<157::AID-AJA8>3.0.CO;2-F 9337136

[8] G. H. Lee , Z. Chhangawala , S. von Daake , et al., “Reelin Induces Erk1/2 Signaling in Cortical Neurons Through a Non‐Canonical Pathway,” Journal of Biological Chemistry 289, no. 29 (2014): 20307–20317. 10.1074/jbc.M114.576249 PMC4106344 24876378

[9] F. Telese , Q. Ma , P. M. Perez , et al., “LRP8‐Reelin‐Regulated Neuronal Enhancer Signature Underlying Learning and Memory Formation,” Neuron 86, no. 3 (2015): 696–710. 10.1016/j.neuron.2015.03.033 PMC4486257 25892301

[10] C. Bosch , N. Masachs , D. Exposito‐Alonso , et al., “Reelin Regulates the Maturation of Dendritic Spines, Synaptogenesis and Glial Ensheathment of Newborn Granule Cells,” Cerebral Cortex 26, no. 11 (2016): 4282–4298. 10.1093/cercor/bhw216 PMC5066826 27624722

[11] H.‐J. Lee , J.‐H. Park , J. H. Trotter , et al., “Reelin and APP Cooperatively Modulate Dendritic Spine Formation In Vitro and In Vivo,” Experimental Neurobiology 32, no. 1 (2023): 42–55. 10.5607/en22044 PMC10017845 36919335

[12] S. Niu , O. Yabut , and G. D'Arcangelo , “The Reelin Signaling Pathway Promotes Dendritic Spine Development in Hippocampal Neurons,” Journal of Neuroscience 28, no. 41 (2008): 10339–10348. 10.1523/JNEUROSCI.1917-08.2008 PMC2572775 18842893

[13] J. T. Rogers , I. Rusiana , J. Trotter , et al., “Reelin Supplementation Enhances Cognitive Ability, Synaptic Plasticity, and Dendritic Spine Density,” Learning & Memory (Cold Spring Harbor, N.Y.) 18, no. 9 (2011): 558–564. 10.1101/lm.2153511 PMC3166788 21852430

[14] U. Beffert , E. Weeber , A. Durudas , et al., “Modulation of Synaptic Plasticity and Memory by Reelin Involves Differential Splicing of the Lipoprotein Receptor Apoer2,” Neuron 47, no. 4 (2005): 567–579. 10.1016/j.neuron.2005.07.007 16102539

[15] S. Qiu , L. F. Zhao , K. M. Korwek , and E. J. Weeber , “Differential Reelin‐Induced Enhancement of NMDA and AMPA Receptor Activity in the Adult Hippocampus,” Journal of Neuroscience: The Official Journal of the Society for Neuroscience 26, no. 50 (2006): 12943–12955. 10.1523/JNEUROSCI.2561-06.2006 PMC6674964 17167084

[16] S. H. Fatemi , J. A. Earle , and T. McMenomy , “Reduction in Reelin Immunoreactivity in Hippocampus of Subjects With Schizophrenia, Bipolar Disorder and Major Depression,” Molecular Psychiatry 5, no. 6 (2000): 654–663. 10.1038/sj.mp.4000783 11126396

[17] Z. Wang , Y. Hong , L. Zou , et al., “Reelin Gene Variants and Risk of Autism Spectrum Disorders: An Integrated Meta‐Analysis,” American Journal of Medical Genetics. Part B, Neuropsychiatric Genetics: The Official Publication of the International Society of Psychiatric Genetics 165B, no. 2 (2014): 192–200, 10.1002/ajmg.b.32222. 24453138

[18] H. Zhang , X. Liu , C. Zhang , et al., “Reelin Gene Alleles and Susceptibility to Autism Spectrum Disorders,” Molecular Psychiatry 7, no. 9 (2002): 1012–1017, 10.1038/sj.mp.4001124. 12399956

[19] E. Costa , J. Davis , D. R. Grayson , A. Guidotti , G. D. Pappas , and C. Pesold , “Dendritic Spine Hypoplasticity and Downregulation of Reelin and GABAergic Tone in Schizophrenia Vulnerability,” Neurobiology of Disease 8, no. 5 (2001): 723–742. 10.1006/nbdi.2001.0436 11592844

[20] D. R. Grayson , X. Jia , Y. Chen , et al., “Reelin Promoter Hypermethylation in Schizophrenia,” Proceedings of the National Academy of Sciences of the United States of America 102, no. 26 (2005): 9341–9346, 10.1073/pnas.0503736102. PMC1166626 15961543

[21] I. Cuchillo‐Ibañez , V. Balmaceda , T. Mata‐Balaguer , I. Lopez‐Font , and J. Sáez‐Valero , “Reelin in Alzheimer's Disease, Increased Levels but Impaired Signaling: When More Is Less,” Journal of Alzheimer's Disease 52 (2016): 403–416, https://journals.sagepub.com/doi/10.3233/JAD‐151193?icid=int.sj‐abstract.citing‐articles.4&utm_source=chatgpt.com. 10.3233/JAD-151193 27031488

[22] D. Seripa , M. G. Matera , M. Franceschi , et al., “The RELN Locus in Alzheimer's Disease,” Journal of Alzheimer's Disease 14, no. 3 (2008): 335–344, 10.3233/jad-2008-14308. 18599960

[23] N. Chen , Y. Bao , Y. Xue , et al., “Meta‐Analyses of RELN Variants in Neuropsychiatric Disorders,” Behavioural Brain Research 332, no. August (2017): 110–119, 10.1016/j.bbr.2017.05.028. 28506622

[24] A. Iemolo , P. Montilla‐Perez , J. Nguyen , V. B. Risbrough , M. A. Taffe , and F. Telese , “Reelin Deficiency Contributes to Long‐Term Behavioral Abnormalities Induced by Chronic Adolescent Exposure to Δ9‐Tetrahydrocannabinol in Mice,” Neuropharmacology 187, no. April (2021): 108495. 10.1016/j.neuropharm.2021.108495 PMC7989547 33582152

[25] T. J. Silva‐Hurtado , G. Giua , O. Lassalle , et al., “Reelin Deficiency and Synaptic Impairment in Adolescent PFC Following Initial Synthetic Cannabinoid Exposure,” Biological Psychiatry: Global Open Science 5 (2024): 100456. 10.1016/j.bpsgos.2024.100426 PMC11804564 39926699

[26] Y. Zuo , N. Formoli , A. Libster , et al., “Single‐Nucleus Transcriptomics Identifies Neuroblast Migration Programs Sensitive to Reelin and Cannabis in the Adolescent Nucleus Accumbens,” bioRxivorg (2025), 10.1101/2025.04.03.646846.

[27] K. L. Brida , E. T. Jorgensen , R. A. Phillips , et al., “Reelin Marks Cocaine‐Activated Striatal Neurons, Promotes Neuronal Excitability, and Regulates Cocaine Reward,” Science Advances 11, no. 13 (2025): eads4441. 10.1126/sciadv.ads4441 PMC12076537 40138397

[28] G. de Guglielmo , A. Iemolo , A. Nur , et al., “Reelin Deficiency Exacerbates Cocaine‐Induced Hyperlocomotion by Enhancing Neuronal Activity in the Dorsomedial Striatum,” Genes, Brain, and Behavior 21, no. 7 (2022): e12828. 10.1111/gbb.12828 PMC9744517 35906757

[29] H. Matsuzaki , Y. Minabe , K. Nakamura , et al., “Disruption of Reelin Signaling Attenuates Methamphetamine‐Induced Hyperlocomotion,” European Journal of Neuroscience 25, no. 11 (2007): 3376–3384. 10.1111/j.1460-9568.2007.05564.x 17553006

[30] B. Escudero , M. Moya , L. López‐Valencia , F. Arias , and L. Orio , “Reelin Plasma Levels Identify Cognitive Decline in Alcohol Use Disorder Patients During Early Abstinence: The Influence of APOE4 Expression,” International Journal of Neuropsychopharmacology 26, no. 8 (2023): 545–556. 10.1093/ijnp/pyad034 PMC10464928 37350760

[31] E. Legaki , N. Dovrolis , N. Moscholiou , et al., “Altered Expression of Neuroplasticity‐Related Genes in Alcohol Addiction and Treatment,” International Journal of Molecular Sciences 25, no. 21 (2024): 11349, 10.3390/ijms252111349. PMC11546795 39518903

[32] J. Le Merrer , J. A. J. Becker , K. Befort , and B. L. Kieffer , “Reward Processing by the Opioid System in the Brain,” Physiological Reviews 89, no. 4 (2009): 1379–1412. 10.1152/physrev.00005.2009 PMC4482114 19789384

[33] G. D'Arcangelo , G. G. Miao , S.‐C. Chen , H. D. Scares , J. I. Morgan , and T. Curran , “A Protein Related to Extracellular Matrix Proteins Deleted in the Mouse Mutant Reeler,” Nature 374, no. 6524 (1995): 719–723. 10.1038/374719a0 7715726

[34] M. Chevée , C. J. Kim , N. Crow , E. G. Follman , M. Z. Leonard , and E. S. Calipari , “Food Restriction Level and Reinforcement Schedule Differentially Influence Behavior During Acquisition and Devaluation Procedures in Mice,” eNeuro 10, no. 9 (2023): ENEURO.0063–ENEU23.2023, 10.1523/ENEURO.0063-23.2023. PMC10537440 37696663

[35] G. Margiani , M. P. Castelli , N. Pintori , et al., “Adolescent Self‐Administration of the Synthetic Cannabinoid Receptor Agonist JWH‐018 Induces Neurobiological and Behavioral Alterations in Adult Male Mice,” Psychopharmacology 239, no. 10 (2022): 3083–3102. 10.1007/s00213-022-06191-9 PMC9481487 35943523

[36] G. Valles , J. L. Huebschman , E. Chow , C. Kelly , Y. Guo , and L. N. Smith , “Jugular Vein Catheter Design and Cocaine Self‐Administration Using Mice: A Comprehensive Method,” Frontiers in Behavioral Neuroscience 16 (2022): 880845. 10.3389/fnbeh.2022.880845 PMC9242005 35783231

[37] D. N. Kearns and S. J. Weiss , “Sign‐Tracking (Autoshaping) in Rats: A Comparison of Cocaine and Food as Unconditioned Stimuli,” Learning & Behavior 32, no. 4 (2004): 463–476. 10.3758/bf03196042 15825887

[38] K. J. Livak and T. D. Schmittgen , “Analysis of Relative Gene Expression Data Using Real‐Time Quantitative PCR and the 2(‐Delta Delta C(T)) Method,” Methods (San Diego, Calif.) 25, no. 4 (2001): 402–408. 10.1006/meth.2001.1262 11846609

[39] C. A. Schneider , W. S. Rasband , and K. W. Eliceiri , “NIH Image to ImageJ: 25 Years of Image Analysis,” Nature Methods 9, no. 7 (2012): 671–675. 10.1038/nmeth.2089 PMC5554542 22930834

[40] A. E. Carpenter , T. R. Jones , M. R. Lamprecht , et al., “CellProfiler: Image Analysis Software for Identifying and Quantifying Cell Phenotypes,” Genome Biology 7, no. 10 (2006): R100. 10.1186/gb-2006-7-10-r100 PMC1794559 17076895

[41] W. Wang , X. Xie , X. Zhuang , et al., “Striatal μ‐Opioid Receptor Activation Triggers Direct‐Pathway GABAergic Plasticity and Induces Negative Affect,” Cell Reports 42, no. 2 (2023): 112089. 10.1016/j.celrep.2023.112089 PMC10404641 36796365

[42] D. S. Rice and T. Curran , “Role of the Reelin Signaling Pathway in Central Nervous System Development,” Annual Review of Neuroscience 24 (2001): 1005–1039, 10.1146/annurev.neuro.24.1.1005. 11520926

[43] S. Bahrami and F. Drabløs , “Gene Regulation in the Immediate‐Early Response Process,” Advances in Biological Regulation 62, no. September (2016): 37–49. 10.1016/j.jbior.2016.05.001 27220739

[44] G. Egervari , J. Landry , J. Callens , et al., “Striatal H3K27 Acetylation Linked to Glutamatergic Gene Dysregulation in Human Heroin Abusers Holds Promise as Therapeutic Target,” Biological Psychiatry 81, no. 7 (2017): 585–594, 10.1016/j.biopsych.2016.09.015. PMC5346335 27863698

[45] N. Gaddis , R. Mathur , J. Marks , et al., “Multi‐Trait Genome‐Wide Association Study of Opioid Addiction: OPRM1 and Beyond,” Scientific Reports 12, no. 1 (2022): 16873. 10.1038/s41598-022-21003-y PMC9546890 36207451

