# Supplementary material for: Reln Haploinsufficiency Enhances Fentanyl‐Induced Locomotion and Dorsal Striatal Activity Without Affecting Opioid Reinforcement and Relapse‐Like Behavior
Source: Genes Brain Behav. 2026 Oct 6;25(5):e70072. doi: 10.1111/gbb.70072 (PMC13641928; doi:10.1111/gbb.70072)
Supplement: Supplementary file 1 [file GBB-25-e70072-s001.docx]

**Supplementary Figures**

**Supplemental Figure 1 | Sex-stratified behavioral responses across fentanyl IVSA phases in WT and *Reln*+/- mice. (A-B)** Active (solid line) and inactive (dashed line) lever presses (A) or infusions (B) across all IVSA sessions (Day 1-15), stratified by sex and genotype. (**C-E)** Total fentanyl intake (ug) averaged across the two sessions at each unit dose during the dose response phase for WT (n=13, black) and *Reln*+/- (n=13, blue) mice (**C**) or separated by females (**D;** WT n=5, Reln+/- n=8) and males (**E;** WT n=8, Reln+/- n=5). Data are mean ± SEM.

**Supplemental Figure 2 | Sex-stratified progressive ratio lever pressing and breakpoint. (A-B)** Total lever presses stratified by sex **(A)** or breakpoint stratified by sex **(B)** during the progressive ratio session. Data are faceted by genotype and sex (female: WT n=5, Reln+/- n=8; male: WT n=8, Reln+/- n=5). Data are mean ± SEM. *p < 0.05.

**Supplemental Figure 3 | Cumulative lever presses for IVSA phases and sex-stratified behavioral and molecular responses across fentanyl extinction and cue-induced fentanyl seeking in WT and *Reln*+/- mice. (A)** Active (solid line) and inactive (dashed line) lever presses during extinction phase (Extinction, Day 1-10), faceted by sex and genotype. **(B)** Comparison of active and inactive lever presses during the first 30 minutes of the last extinction session (Ext., Day 10) and during the subsequent 30 minute cue-induced fentanyl seeking session, faceted by genotype and sex. **(C-D)** Cumulative summation of lever presses per each IVSA phase **(C)**, stratified by sex **(D)**. In (C) and (D), active lever presses are shown as solid lines and inactive lever presses as dashed lines. Data are faceted by genotype and/or sex (female: WT n=5, Reln+/- n=8; male: WT n=8, Reln+/- n=5). Data are mean ± SEM. *p < 0.05.

**Supplemental Figure 4 | Sex-stratified IEG RT-qPCR for naive and cue-induced fentanyl seeking in WT and *Reln*+/- mice. (A)** Relative expression (2^-ΔCt) of *Fos, Arc, Egr1,* and *Npas4* expression in the dorsal striatum from naive mice from the homecage, normalized to Gapdh; faceted by genotype. **(B)** Relative expression (2^-ΔCt) of *Fos, Arc, Egr1,* and *Npas4* expression in the dorsal striatum following cue-induced fentanyl seeking, normalized to Gapdh; faceted by sex and genotype. **(C)** Relative expression (2^-ΔCt) of *Fos, Arc, Egr1,* and *Npas4* expression in the dorsal striatum from naive mice from the homecage, normalized to Gapdh; faceted by sex and genotype. Data are mean ± SEM (cue-induced fentanyl seeking: Female: WT n=5, *Reln*+/- n=8; Male: WT n=8, *Reln*+/- n=5 - Naive: Female: WT n=3, *Reln*+/- n=3; Male: WT n=4, *Reln*+/- n=3).

**Supplemental Figure 5 | Daily locomotion during CPP conditioning and sex-stratified fentanyl CPP analysis. (A)** Absolute time spent (s, seconds) per saline- or fentanyl-paired room for each dose stratified by genotype and sex. **(B)** Change in percent time spent in the fentanyl-paired room on the test day relative to baseline, for mice conditioned at 20 µg/kg or 80 µg/kg, faceted by sex and genotype. (**C)** Average locomotor speed (total distance / total time) during the pretest or first CPP conditioning session following acute fentanyl injection at 20 or 80 µg/kg, faceted by genotype and sex. **(D)** Average locomotor speed (total distance / total time) for acute fentanyl injection at 20 or 80 µg/kg or saline control when all days per treatment all averaged, faceted by genotype. **(E-F)** Locomotion for pretest or fentanyl/saline CPP conditioning days across all days faceted by genotype **(E)** and sex **(F)**. Data are mean ± SEM. Locomotion, baseline, and conditioning: Female - WT n=4; Reln+/- n=4; Male - WT n=4, Reln+/- n=4 per each dose. Test Day: Female - WT n=4; Reln+/- n=4; Male - WT n=4, Reln+/- n=3 for low dose and WT n=4; Reln+/- n=4; Male - WT n=4, Reln+/- n=4 for high dose.

**Supplementary Table**

**Supplementary Table 1. Fentanyl unit-dose delivery parameters for intravenous self-administration.**

**Supplementary Table 2. Primer sequences used for RT-qPCR.** Forward and reverse primer sequences (5′→3′) for each target immediate early gene (*Fos, Arc, Egr1, Npas4*) and the *Gapdh* housekeeping gene used for normalization.

**Supplementary Table 3.01-3.14. Statistical results for fentanyl IVSA behavior.** Outputs for each IVSA phase (acquisition, dose-response, dose reset, progressive ratio, extinction, and cue-induced fentanyl seeking) modeling lever presses, infusions, intake, and breakpoint.

**Supplementary Table 4.01-4.02. Statistical results for dorsal striatal RT-qPCR**. Linear-model outputs for immediate early gene expression (*Fos, Arc, Egr1, Npas4*) following cue-induced fentanyl seeking (4.01) and in naïve mice (4.02), with genotype and sex as fixed effects and post hoc estimated marginal means comparisons.

**Supplementary Table 5.01-5.04. Statistical results for conditioned place preference and fentanyl-induced locomotion.** Outputs for absolute chamber occupancy (5.01), Δ% preference (5.02), and fentanyl-induced locomotion, comparing baseline versus fentanyl conditioning day 1 (5.03) and saline versus fentanyl across conditioning days (5.04), each with genotype, dose, treatment, and/or sex as fixed effects and post hoc estimated marginal means comparisons.

**Supplementary Table 6. Statistical results for dorsal striatal Fos immunoreactivity.** Linear mixed model output for the percentage of Fos+/DAPI+ cells, with genotype as a fixed effect, mice and nested slices as random effects, and post hoc estimated marginal means comparisons.
